# Tendon Disorders in Chronic Liver Disease: A Retrospective Cohort Study in Taiwan

**DOI:** 10.3390/ijerph20064983

**Published:** 2023-03-12

**Authors:** Ching-Yueh Lin, Shih-Chung Huang, Shiow-Jyu Tzou, Chun-Hao Yin, Jin-Shuen Chen, Yao-Shen Chen, Shin-Tsu Chang

**Affiliations:** 1 Department of Physical Medicine and Rehabilitation, Kaohsiung Armed Forces General Hospital, Kaohsiung 802301, Taiwan; 2 Department of Physical Medicine and Rehabilitation, Tri-Service General Hospital, School of Medicine, National Defense Medical Center, Taipei 114202, Taiwan; 3 Division of Cardiology, Department of Internal Medicine, Kaohsiung Armed Forces General Hospital, Kaohsiung 802301, Taiwan; 4 Institute of Medical Science and Technology, National Sun Yat-sen University, Kaohsiung 804201, Taiwan; 5 Division of Cardiology, Department of Internal Medicine, Tri-Service General Hospital, National Defense Medical Center, Taipei 114202, Taiwan; 6 Teaching and Researching Center, Kaohsiung Armed Forces General Hospital, Kaohsiung 802301, Taiwan; 7 Department of Medical Education and Research, Kaohsiung Veterans General Hospital, Kaohsiung 813414, Taiwan; 8 Institute of Health Care Management, National Sun Yat-sen University, Kaohsiung 804201, Taiwan; 9 Department of Administration, Kaohsiung Veterans General Hospital, Kaohsiung 813414, Taiwan; 10 Department of Physical Medicine and Rehabilitation, Kaohsiung Veterans General Hospital, Kaohsiung 813414, Taiwan

**Keywords:** betel nut, chronic liver disease, glucocorticoids, statins, tendinopathy, tendon disorder

## Abstract

To investigate the relationship between chronic liver disease and tendon disorder, a retrospective cohort study was conducted using the Kaohsiung Veterans General Hospital database. Patients >18 years with newly diagnosed liver disease and with at least a two-year follow-up in the hospital were included. An equal number of 20,479 cases were enrolled in both the liver-disease and non-liver-disease groups using a propensity score matching method. Disease was defined using ICD-9 or ICD-10 codes. The primary outcome was the development of tendon disorder. Demographic characteristics, comorbidities, use of tendon-toxic drugs, and status of HBV/HCV infection were included for analysis. The results showed 348 (1.7%) and 219 (1.1%) individuals developed tendon disorder in the chronic liver disease group and non-liver-disease group. Concomitant use of glucocorticoids and statins may have further raised the risk of tendon disorder in the liver disease group. The co-existence of HBV/HCV infection did not increase the risk of tendon disorder in the patients with liver disease. Considering these findings, physicians should be more aware of tendon issues in advance, and a prophylactic strategy should be adopted in patients with chronic liver disease.

## 1. Introduction

Tendinitis, ligament injury, bursitis, and fasciitis are common musculoskeletal complaints, which often cause pain, functional impedance, and lower quality of life for injured individuals [1]. Since the 1990s, surgical biopsies have become prevalent, and the use of “tendinitis” was replaced by the use of “tendinopathy” or “tendinosis”, based on a histopathology that essentially presented a degenerative condition caused by repetitive mechanical loading rather than an inflammatory reaction in most cases [2]. However, a systemic review in 2018 concluded that an inflammatory response does occur in tendinopathy cases, even in the chronic stage [3]. Biomechanically, it was found that vascular injuries in the tendon ignited a minimal inflammation; recruited monocytes, macrophages, lymphocytes, but less neutrophils; and then increased COX-2, IL-6 expression, and PGE2 production. Histologically, this featured a scarring and healing reaction and was called “angiofibroblastic hyperplasia” [2]. Risk factors for overuse of tendinopathy included intrinsic factors, such as age older than 35 years, gender (e.g., estrogen level and menopause, biomechanical variables, such as hip-to-knee angles), biomechanical abnormalities (e.g., pes planus, joint stiffness, muscle tightness), and genetics and heritability (e.g., single nucleotide polymorphism related low GDF-5 level, rheumatic disease); and extrinsic factors, such as training error, poor environment, and equipment setting [2].

Recently, increasing attention has been given to the relationship between metabolic disease and tendon health. For instance, diabetic populations are prone to develop tendinopathy at various sites and the condition is also more ominous. Hypercholesterolemia, hypertriglyceridemia, and hyperuricemia are also risk factors for tendinopathy [2]. Chronic kidney patients had a worse prognosis for disease of the cuff tendon or other musculoskeletal organ [4,5]. A recent study by Lin et al. [6] also demonstrated a higher risk of cuff tendon disease in patients with chronic liver disease.

Chronic liver disease covers a broad spectrum of liver conditions [7], e.g., chronic hepatitis associated with hepatitis B virus (HBV) and hepatitis C virus (HCV) infection; non-alcoholic fatty liver disease (NAFLD), alcoholic liver disease, and non-alcoholic and alcoholic steatohepatitis; primary biliary cholangitis (PBC) and primary sclerosing cholangitis (PSC), in which only PSC has the pre-malignancy feature; autoimmune hepatitis that is correlated with autoimmune system, but also genetic- and environmental- related; and cardiac system-related congestive hepatopathy. The etiologies are heterogeneous and multifaceted, ranging from biomechanical, cellular, metabolic, genetic factors, microbiota, behavioral, to environmental interactions. The new nomenclature of metabolic dysfunction associated-fatty liver disease (MAFLD) has been suggested to replace NAFLD, because it emphasizes the relationship of the disease with metabolic dysfunction [8]. It defines the disease with evidence of hepatosteatosis using any one of three criteria: obesity, type 2 diabetes mellitus, and metabolic dysregulation. Aside from NAFLD [8,9], metabolic dysfunction is commonly found in other liver diseases, such as alcoholic liver disease [10] and chronic hepatitis C [11]. As time passes, liver disease can progress to liver cirrhosis, an end-stage liver conditions with permanent scarring and damage to the liver. The severity is classified using the modified Child–Pugh classification scale, which is based on the serum bilirubin and albumin, the prothrombin time, the severity of ascites, and the grade of encephalopathy.

Due to the particularly high prevalence of chronic liver disease in Taiwan [12] and its association with metabolic dysfunction, this cohort study aimed to investigate the relationship between chronic liver disease and tendon disorder. Additionally, the study evaluated the impact of certain drugs on tendon health in this population.

## 2. Materials and Methods

The clinical database of the Kaohsiung Veterans General Hospital (KSVGH) with a registry of 753,544 consecutive outpatients from January 2013 to March 2020 was used. Demographic data, including sex, age, cigarette use, alcohol use, betel nut use, and medical records, were collected from the database. Diagnoses and comorbidities were defined using the disease codes from the International Classification of Diseases, Ninth and Tenth Revision, Clinical Modification (ICD-9-CM and ICD-10-CM). (Table S1) Drug codes obtained from the National Health Health Insurance Administration website were used to identify the use of relevant medications. The study design was approved by the KSVGH Committee on Human Research (KSVGH20-CT7-16), in which the need for informed patient consent was waived because of the use of de-identified patient data. This study was conducted in compliance with the Declaration of Helsinki (1964).

### 2.1. Inclusion and Exclusion Criteria

Patients with newly registered codes for liver disease, in the hospital as outpatients, ranked in the first three at least twice, and with disease at follow-up after at least two years were enrolled from the clinical database of KSVGH during Jan 2013–March 2020. Exclusion criteria included any event of tendon disorder before the index date and age <18. Patients who met the inclusion and exclusion criteria entered the liver-disease group.

As a comparison group, patients without diagnosis of liver disease and who met the exclusion criteria were matched before entry. Propensity score matching was used, in a ratio of 1:1, adjusted by age, gender, index year, and relevant comorbidities, including hypertension, diabetes, chronic kidney disease, and thyroid disease. The index year was the year of first registration of codes for liver disease.

### 2.2. Comorbidities

Comorbidities, including thyroid disease, diabetes, dyslipidemia, gout, depression, hypertension, ischemic heart disease, heart failure, chronic kidney disease, connective-tissue disease, and osteoporosis, were considered potential risk factors for tendon disorder.

### 2.3. Tendon-Toxic Medications

Fluoroquinolone, glucocorticoids, aromatase inhibitors, and 3-hydroxy-3-methyl-glutaryl-coenzyme A (HMG-CoA) reductase inhibitors (i.e., statins) are four categories that can lead to tendinopathy [13]. Prescriptions of these drugs from three months prior to the index date (the date of first registration of liver disease) until three months prior to the onset of event or before the endpoint of the study were recorded and analyzed in the liver-disease group. ATC codes were used to define the use of drugs (H02AB is systemic glucocorticoids; C10AA is statins; J01MA is fluoroquinolone; L02BG is aromatase inhibitors). A cumulative dose, defined as the sum of days, was used to evaluate the side effect of these drugs on tendons. Cumulative doses of glucocorticoids ≥30 days [14], statins ≥90 days [15], fluoroquinolone ≥7 days [16,17], and aromatase inhibitors ≥30 days [18,19] were the thresholds that we assumed had a clinical tendon-toxic effect, based on a literature review and empirical experience.

### 2.4. Laboratory Tests for HBV and HCV

To examine the effect of viral hepatitis on tendon disorder in the liver-disease group, the results of HBs-Ag and anti-HCV tests from patients with chronic liver disease were extracted and analyzed in the subgroup analysis if data were available.

### 2.5. Outcome Measures

The outcome measure was defined as any event of tendon disorder that occurred after the index date. Liver diseases were divided into two categories, with or without liver cirrhosis, defined using the ICD-9 or ICD-10 codes (Table S1). Tendon disorder was defined using the ICD-9 or ICD-10 codes, encompassing tendonitis, synovitis, bursitis, and capsulitis, at various sites from the arm, forearm, thigh, to leg; and with different severities, from inflammation to complete tear (Table S1).

### 2.6. Statistical Analysis

This matched study was conducted using propensity score matching at a ratio of 1:1, matched by age, gender, index year, and relevant comorbidities, to minimize the effect of confounding factors. Propensity scores provide a method for matching using multiple confounding variables without limitations of the covariate matching method [20]. The baseline demographic data, characteristics, and comorbidities of the patients were compared between the liver-diseased and non-liver-diseased groups. Categorical variables were analyzed using a Pearson chi-square test and described as proportions, and continuous variables were compared using one-way analysis of variance and expressed as mean ± SD.

Univariable conditional logistic regression was used to estimate the crude odds ratio (OR) and 95% confidence interval (CIs) of each variable, to evaluate its effect on the tendon event, then any variable with a significance at *p* < 0.10 was selected as a candidate for the multivariable conditional logistic regression [21]. A forest plot was used to systematically represent the association among significant variables based on the multivariable conditional logistic regression. The result was statistically significant with a *p*-value <0.05. SAS software (SAS System for Windows, version 9.2; SAS Institute, Cary, NC, USA) and SPSS statistical software 22.0.0 (IBM Corp., Armonk, NY, USA) were used to perform all statistical analyses.

## 3. Results

**Figure 1:**
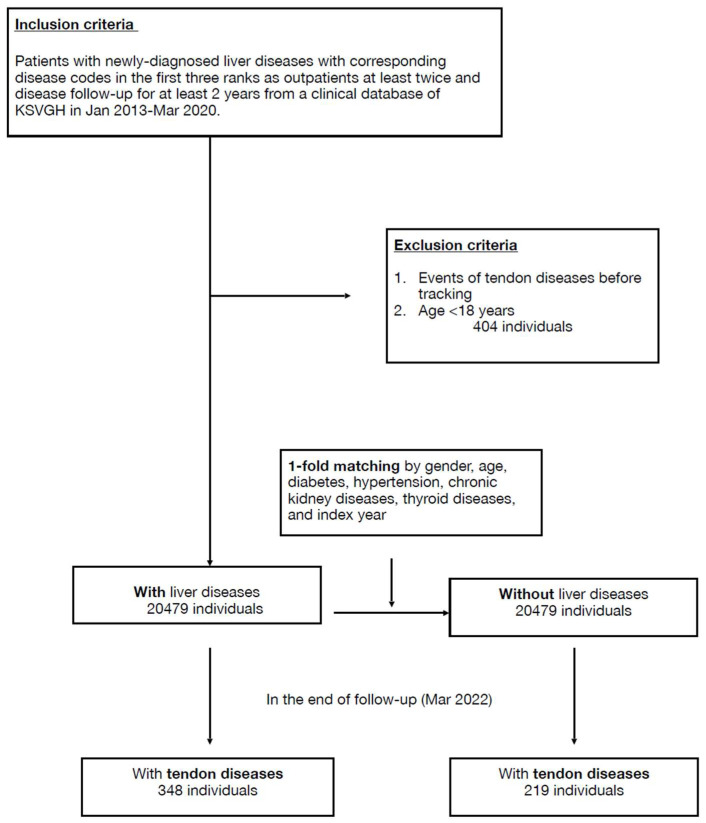
Flowchart.

Initially, 21,810 cases with newly diagnosed liver diseases from the clinical database of KSVGH during January 2013–March 2020 were enrolled, and then 404 cases were excluded due to age <18 or coexisting tendon disease. After 1-fold matching adjusted by age, sex, and specified comorbidities, 20,479 cases were assigned to both the liver-disease and non-liver-disease groups (Figure 1).

**Figure 2:**
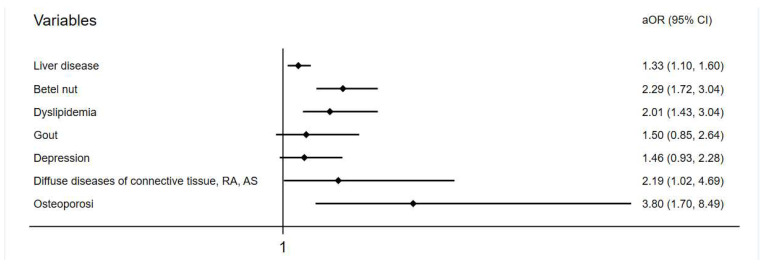
Adjusted odds ratio of variables in a forest plot.

Baseline demographic data of both groups are shown in the Table 1. At the endpoint, 348 (1.7%) and 219 (1.1%) individuals developed tendon diseases in the liver-disease and comparison groups, respectively. The incidence of tendon disease was higher in the liver-disease group, with statistical significance (*p* < 0.001) (Table 2). The mean follow-up duration in the study was 73.7 ± 25.5 months for the liver-disease group and 75.11 ± 26.0 months for the comparison group. The average onset of 39 months for tendon disorder in the liver-disease group was slightly longer than the 33 months of the comparison group but was not significant (Table S2). After multivariate conditional logistic regression, the adjusted odds ratio and 95% confidence interval for tendon disease were 1.33 (1.10–1.60) with liver disease, 2.29 (1.72–3.04) with chewing betel nut, 2.01 (1.34–3.04) having dyslipidemia, 2.19 (1.02–4.69) having connective tissue disease, and 3.80 (1.70–8.49) having osteoporosis (Table 3, Figure 2).

In the subgroup analysis, a higher incidence of tendon disease was noted with concurrent use of glucocorticoids ≥ 30 days, statins ≥ 90 days, and aromatase inhibitors ≥ 30 days. Statistical significance was only shown for the use of glucocorticoids and statins (Table 4). In total, 8940 out of 20,479 patients were coded with liver cirrhosis (43.7%). The incidence of tendon disorder was higher in those with codes for cirrhosis compared with those without (2.1% vs. 1.4%) (Table S3). The incidence of tendon disorder showed no difference regarding the status of HBV/HCV infection (Table S4).

## 4. Discussion

In our study, chronic liver disease raised the risk of tendon disorder to around 1.33-fold higher than usual. Tendon disorder occurred on average three years after being diagnosed with liver disease. The risk of tendon disorder increased further in this population with concurrent use of certain tendon-toxic medications, such as systemic glucocorticoids and statins. Finally, status of HBV/HCV infection did not modify the risk of tendon disorder in this population.

Viral hepatitis and its chronic complications have had a heavy social burden in Taiwan, and the particularly high prevalence is mainly attributed to the high transmission of HBV and HCV via a longitudinal pathway or blood products. In addition, habitual large-quantity alcohol consumption is common in certain populations and social occasions in Taiwan, resulting in a high prevalence of alcoholic liver disease and cirrhosis. In the past decades, investigators have established a strong relationship between liver disease and joint disorder, from the perspectives of an impaired complement system and [22] and increased serum gammaglobulin [23]. Both studies [24,25] illustrated common rheumatic conditions in liver diseases. Another review [26] specifically discussed rheumatoid arthritis- and anti-rheumatic drug-associated liver dysfunction. A recent study proved that people with chronic liver illness were vulnerable to internal joint derangement [27]. It suggested that this result was in accordance with the Traditional Chinese Medicine theory of “Liver governs tendon” [27], where the liver controls tendon health, and the tendon health consequently decides joint health. Our previous study also directly showed that the risk of cuff tendon tear increased with chronic liver disease [6]. These findings explained the tendon pathology from the perspective of immuno-dysregulation.

Metabolic diseases also profoundly interfered with tendon health, including type 2 diabetes mellitus, dyslipidemia, chronic kidney disease, and so on [2,4,5]. In our result, dyslipidemia was an independent risk factor for tendon disorder, consistent with previous studies [28,29]. As the lipid profile became abnormal, tendon pathology might form via xanthoma formation, alterations in tenocyte gene and protein expression, disorganized matrix turnover, and cytokine dysregulation [28,29]. The use of hypercholesterolemia drugs, e.g., statins, can also accelerate the pathologic process, although the mechanism is unclear [29]. In liver disease, NAFLD was found to be caused by metabolic dysfunction; conversely, overproduction of triglycerides and glucose in the fatty liver increased metabolic syndrome [9]. Similar vicious cycles occur in other liver diseases, such as alcoholic liver disease [10] and chronic hepatitis C [11].

Another important issue is Vitamin D deficiency in chronic liver disease. In a review, Dougherty et al. illustrated the crosstalk between calcium, vitamin D, and parathyroid hormone, and its role in muscle health, bone formation, and tendon-to-bone healing [30]. Vitamin D positively affected anti-inflammatory response; bone strengthening by regulating nuclear factor-κB (NF-κB) (RANK) and BMPs; muscle healing and functioning through myocyte regulation; and tendon-to-bone healing, through regulating matrix metalloproteinases (MMPs) and tissue inhibitors of MMPs (TIMPs). Vitamin D level was usually low in chronic liver disease, even in a milder forms of the disease [31]. If glucocorticoids were indicated and used in these patients, the Vitamin D level could be further reduced. A large national cross-sectional survey [32] and a systematic review [33] both verified the association of glucocorticoid use and low Vitamin D level. The mechanism of the enhanced transcription of 24(OH)ase by glucocorticoids and the induced catabolism of Vitamin D was clarified in an in vitro study [34]. Other common comorbidities in liver diseases can also account for tendon illness; for example, an unbalanced immune system, pro-inflammatory cytokines, hypogonadism, elevated bilirubin levels, and interacting pharmacological effects [31]. Broadly speaking, the etiologies of tendon disorder in chronic liver disease are diverse and multifaceted, involving inflammatory cytokines and the immune system, the calcium–vitamin D-parathyroid hormone axis, metabolic homeostasis, drugs, social and environmental interactions, and so forth.

Betel nut chewing has been widely validated a hazardous practice in causing various liver diseases [35], ranging from chronic liver disease, liver fibrosis, liver cirrhosis (LC), to hepatocellular carcinoma (HCC). Betel nut chewing was not only an independent risk of developing LC and HCC, but also additive for enhancing liver complications related to HBV and HCV [36]. Betel nut chewing also carried a higher risk of liver fibrosis in certain comorbidities, e.g., non-alcoholic fatty liver disease [37] and metabolic syndrome [38]. In addition, betel nut chewing was related to a higher incidence of increased arterial stiffness, hypertension, dyslipidemia, metabolic syndrome, diabetes, and cardiovascular disease [39]. So far, there is lack of evidence showing that betel nut chewing is harmful to tendon health; however, we suggest that betel nut chewing may lead to liver disease, metabolic syndrome, and systemic arterial stiffness, all of which subsequently undermine the tendon integrity. After adjusting for variables, betel nut chewing remained a significant risk factor for developing tendon disorder.

Osteoporosis was an independent risk factor for tendon disorder in our results. Low bone marrow density (BMD) was found to increase cuff tendon re-tear and revision rate following repair surgery [40]. In another study, the authors used direct bone density measurement to validate a significant association between low bone density and cuff tendon disorders, presuming that the bone density was already decreased before the tendon tear [41]. The above findings support that osteopenia contributed to the poor tendon quality, as in the findings in our study. The literature has also reported that osteopenia and sarcopenia are commonplace in various liver diseases. The receptor activator of nuclear factor kappa (RANK)-RANK ligand-osteoprotegerin (OPG) system and pro-inflammatory cytokines, metabolite imbalance such as bilirubin, sclerostin, and insulin-like growth factor-1 accounted for osteoporosis, while insulin resistance and obesity in NAFLD and hyperammonemia, low branched-chain amino acids, and hypogonadism in liver cirrhosis resulted in sarcopenia in liver disease. The bilateral crosstalk between the muscle and bone through myostatin, irisin, β-aminoisobutyric acid (BAIBA), osteocalcin, and the RANK and the Wnt/β-catenin pathways was also responsible for the formation of osteosarcopenia [42]. Given the above knowledge about bone and muscle health, we should be more alert to tendon issues in chronic liver disease patients who have concurrent osteoporosis and sarcopenia. Soft-tissue rheumatic disorders (ICD9, 10: connective-tissue-disorders) are commonly related to soft-tissue rheumatic conditions, such as tendinitis, in accordance with our results [1].

We studied the side effects of certain drugs on tendons in liver disease patients in the subgroup analysis, and a cumulative treatment of glucocorticoids and statins yielded a negative and significant effect, and cumulative use of aromatase inhibitors showed a negative effect, although insignificant. Knobloch summarized that four main categories of drugs, corticosteroids, chinolon antibiotics (e.g., fluoroquinolone), aromatase inhibitors, and statins, were potentially tendon-toxic, by causing histologic changes and biomechanical weakness in the tendon [13]. The average onset of tendon symptoms after starting drug use and the real extent of tendon injury widely varied, dependent on physical health, types of medications, indications, and the interaction of drugs [14,15,16,17,18,19].

A strength of the present study was the use of a large hospital cohort of newly diagnosed chronic liver disease patients with at least a 2-year follow-up to examine the incidence rate and average onset time of tendon disorder. Considering the common situation of the use multiple medications in patients with chronic liver disease, we analyzed certain tendon–toxic drug interactions in the subgroup analysis. We also evaluated if the HBV/HCV infection had an impact on the incidence of tendon disorder in the liver-disease patients.

However, there are some limitations. First, the data were obtained from a hospital database in a medical center, so the related medical history before the wash-out and study period and medical visits outside the hospital were not available, which may have caused information bias. Second, the sample was collected from a tertiary-care hospital, which is expected to have a higher percentage of patients with higher disease severity and comorbidities, so the external generalizability was restricted. Third, laboratory data and image information, which are crucial to determine liver disease severity, were not fully available in the current database, which limited the study to analyzing the impact of different disease severities. Instead, the ICD-9 and ICD-10 codes were used to divide liver diseases into categories regarding cirrhosis status, but these codes, alone, were insufficient to reflect the real disease severity. Lastly, we were unable to predict to what extent liver disease undermined tendon health in the pre-clinical stage. The actual bio-physiology related to tendon pathology in chronic liver disease remains unclear. Further studies including larger sample sizes in different settings and with a longer follow-up period are needed, to clarify the actual etiology and causal relationships.

## 5. Conclusions

On average, patients with chronic liver disease were more likely to develop tendon disorder 39 months after being diagnosed with liver disease compared with the control group. Cumulative use of systemic glucocorticoids and statins were independent risk factors for tendon disorder in the liver disease group. The presence of HBV/HCV did not imply worse tendon outcomes in the liver disease group. This study delivers important messages for physicians, to be aware of tendon issues in such patients in advance and to be more cautious with the prescription of certain drugs, such as glucocorticoids and statins. Health policy campaigns to reduce betel nut use may also be helpful for the overall health in such patients.

## Figures and Tables

**Table 1:** Demographic characteristics at baseline.

	Liver-Disease	Non-Liver-Disease	
Variables	*n* = 20,479 (%)	*n* = 20,479(%)	*p*-value
Demographics			
Sex			0.609
Male	11,969 (58)	12,020 (59)	
Female	8510 (42)	8459 (41)	
Age (year)			0.138
18–39	3122 (15)	3079 (15)	
40–59	9543 (47)	9390 (46)	
≥60	7814 (38)	8010 (39)	
Cigarette	4312 (21)	3291 (16)	<0.001
Alcohol	3525 (17)	2487 (12)	<0.001
Betel nut	11,691 (57)	8760 (43)	<0.001
Comorbidities			
Thyroid disease	510 (2)	496 (2)	0.655
Diabetes	2743 (13)	2860 (12)	0.092
Dyslipidemia	2691 (13)	1724 (8)	<0.001
Gout	949 (5)	534 (3)	<0.001
Depression	331 (2)	172 (1)	<0.001
Hypertension	4605 (23)	4720 (23)	0.175
Ischemic heart disease	1666 (8)	1728 (8)	0.266
Heart failure	497 (2)	480 (2)	0.582
Chronic kidney disease	630 (3)	507 (2)	<0.001
Connective-tissue disease	755 (4)	276 (1)	<0.001
Osteoporosis	525 (3)	309 (2)	<0.001

**Table 2:** Outcomes at the endpoint.

	Liver-Disease	Non-Liver-Disease	
Variables	*n* = 20,479 (%)	*n* = 20,479(%)	*p*-value
Tendon disease/rupture			<0.001
Yes	348 (2)	219 (1)	
No	20,131 (98)	20,260 (99)	

**Table 3:** Outcomes according to conditional logistic regression model.

	OR	95% CI	*p*-Value	aOR	95% CI	*p*-Value
Liver disease						
With	1.59	1.34–1.88	<0.001	1.33	1.10–1.60	0.003
Without		Ref			Ref	
Gender						
Male	0.5	0.15–1.66	0.258			
Female		Ref				
Smoking	1.28	0.92–1.78	0.137			
Drinking	1.27	0.89–1.81	0.181			
Betel nut	2.58	1.97–3.38	<0.001	2.29	1.72–3.04	<0.001
Comorbidity						
Thyroid diseases	1.57	0.61–4.05	0.35			
Diabetes	1.25	0.34–4.66	0.739			
Dyslipidemia	2.31	1.61–3.32	<0.001	2.01	1.34–3.04	0.001
Gout	1.95	1.15–3.30	0.013	1.5	0.85–2.64	0.166
Depression	2.33	0.90–6.07	0.082	1.46	0.93–2.28	0.098
Hypertension	0.5	0.15–1.66	0.258			
Ischemic heart disease	1.79	1.20–2.66	0.004			
Heart failure	0.78	0.29–2.09	0.618			
Chronic kidney disease	1	0.32–3.10	1			
Connective-tissue disease	3.3	1.63–6.70	0.001	2.19	1.02–4.69	0.045
Osteoporosis	4	1.93–8.30	<0.001	3.8	1.70–8.49	0.001

**Table 4:** The association of drug exposure and development of tendon disease.

Drug Exposure	Eventful, *n* (%)	Non-Eventful, *n* (%)	*p*-Value
Glucocorticoids ≥ 30 days	85 (15)	3547 (9)	<0.001
Statins ≥ 90 days	6 (1)	178 (0)	0.029
Fluoroquinolones ≥ 7 days	24 (4)	2157 (5)	0.243
Aromatase inhibitors ≥ 30 days	45 (8)	2477 (6)	0.076

## Data Availability

The clinical database of Kaohsiung Veterans General Hospital (KSVGH) used to support the findings of this study is restricted by the Kaohsiung Veterans General Hospital Committee on Human Research (KSVGH20-CT7-16), in order to protect patient privacy. Data are available from Research Center of Medical Informatics of Kaohsiung Veterans General Hospital, ksnhird@vghks.gov.tw for researchers who meet the criteria for access to confidential data.

## Supplementary Materials

The following supporting information can be downloaded at: https://www.mdpi.com/article/10.3390/ijerph20064983/s1, Table S1: ICD-9/ICD-10 codes; Table S2: Total length of follow-up in the eventful and non-eventful cases in both groups; Table S3. The proportion of tendon disorders in liver disease by the codes for liver cirrhosis; Table S4: The distribution of viral hepatitis among patients with tendon disorder in the liver-disease group.
