# Supplementary material for: Tendon Disorders in Chronic Liver Disease: A Retrospective Cohort Study in Taiwan
Source: Int J Environ Res Public Health. 2023 Mar 12;20(6):4983. doi: 10.3390/ijerph20064983 (PMC10049230; doi:10.3390/ijerph20064983)
Supplement: Supplementary file 1 [file ijerph-20-04983-s001.zip › Table_S1.pdf]

Table S1. ICD-9/ICD-10 codes.

	ICD9		ICD10	
Liver disease	571.4, 571.8, 571.9	Chronic hepatitis	K73	Chronic hepatitis
	570, 571.0-571.1, 571.3, 573	Other liver diseases	K70.0-70.1, K70.4, K70.9, K71.0-71.6, K71.8-71.9, K72, K75, K76, K77	Other liver diseases
	571.2, 571.5-571.6,	Cirrhosis of liver	K70.2-70.3, K71.7, K74	Cirrhosis of liver
Comorbidity	240-246	Disorders of thyroid	E00-E07	Disorders of thyroid
	250	Diabetes	E10-11	Type 1, Type 2 diabetes mellitus
	272	Dyslipidemia	E78	Dyslipidemia
	274	Gout	M10	Gout
	296.2-296.3, 296.82, 300.4, 311	Depression	F32-F33	Major depressive disorder, single episode/recurrent
	401-405	Hypertension	I10-I16	Hypertensive diseases
	410-414	Ischemia heart disease	I20-I25	Ischemic heart disease
	428	Heart failure	I50	Heart failure
	585	Chronic Kidney Disease	N18	Chronic kidney disease
	710, 714, 720	Diffuse diseases of connective tissue, RA, AS	M05, M30-M36, M45	Rheumatoid arthritis with rheumatoid factor; systemic involvement of connective tissue; Ankylosing spondylitis
	733	Osteoporosis	M80-M85	Disorders of bone density and structure
Tendon disorder				
Arm	726	Adhesive capsulitis of shoulder	M75.0	Adhesive capsulitis of shoulder

	726.1	Rotator cuff syndrome of shoulder and allied disorders	M75.2	Bicipital tendinitis
	726.1	Disorders of bursae and tendons in shoulder region, unspecified	M75.3	Calcific tendinitis of shoulder
	726.11	Calcifying tendinitis of shoulder	M75.4	Impingement syndrome of shoulder
	726.12	Bicipital tenosynovitis	M75.5	Bursitis of shoulder
	727.61	Complete rupture of rotator cuff	M65.81	Other synovitis and tenosynovitis, shoulder
	727.62	Rupture of tendons of biceps (long head)	M65.82	Other synovitis and tenosynovitis, upper arm
			M75.1	Rotator cuff tear or rupture, not specified as traumatic
			M66.21	Spontaneous rupture of extensor tendons, shoulder
			M66.31	Spontaneous rupture of flexor tendons, shoulder
			M66.81	Spontaneous rupture of other tendons, shoulder
			M66.22	Spontaneous rupture of extensor tendons, upper arm
			M66.32	Spontaneous rupture of flexor tendons, upper arm

			M66.82	Spontaneous rupture of other tendons, upper arm
Forearm	726.3	Enthesopathy of elbow region	M65.3	Trigger finger
	726.4	Enthesopathy of wrist and carpus	M65.4	Radial styloid tenosynovitis
	727.03	Trigger finger (acquired)	M65.84	Other synovitis and tenosynovitis, hand
	727.04	Radial styloid tenosynovitis	M72.0	Palmar fascial fibromatosis [Dupuytren]
	727.05	Other tenosynovitis of hand and wrist	M77.0	Medial epicondylitis
	727.63	Rupture of extensor tendons of hand and wrist	M77.1	Lateral epicondylitis
	727.64	Rupture of flexor tendons of hand and wrist	M65.83	Other synovitis and tenosynovitis, forearm
			M66.23	Spontaneous rupture of extensor tendons, forearm
			M66.33	Spontaneous rupture of flexor tendons, forearm
			M66.83	Spontaneous rupture of other tendons, forearm
			M66.24	Spontaneous rupture of extensor tendons, hand
			M66.34	Spontaneous rupture of flexor tendons, hand
			M66.84	Spontaneous rupture of other tendons, hand

Thigh	726.5	Enthesopathy of hip region	M65.85	Other synovitis and tenosynovitis, thigh
	727.65	Rupture of quadriceps tendon	M66.25	Spontaneous rupture of extensor tendons, thigh
			M66.35	Spontaneous rupture of flexor tendons, thigh
			M66.85	Spontaneous rupture of other tendons, thigh
Leg	726.6	Enthesopathy of knee	M65.86	Other synovitis and tenosynovitis, lower leg
	726.7	Enthesopathy of ankle and tarsus, unspecified	M65.87	Other synovitis and tenosynovitis, ankle and foot
	726.71	Achilles bursitis or tendinitis	M66.26	Spontaneous rupture of extensor tendons, lower leg
	726.72	Tibialis tendinitis	M66.36	Spontaneous rupture of flexor tendons, lower leg
	727.06	Tenosynovitis of foot and ankle	M66.86	Spontaneous rupture of other tendons, lower leg
	727.66	Rupture of patellar tendon	M66.27	Spontaneous rupture of extensor tendons, ankle and foot
	727.67	Rupture of achilles tendon	M66.37	Spontaneous rupture of flexor tendons, ankle and foot
	727.68	Rupture of other tendons of foot and ankle	M66.87	Spontaneous rupture of other tendons, ankle and foot

	M72.2	Plantar fascial fibromatosis
	M76.6	Achilles tendinitis
