# Supplementary material for: Tendon Disorders in Chronic Liver Disease: A Retrospective Cohort Study in Taiwan
Source: Int J Environ Res Public Health. 2023 Mar 12;20(6):4983. doi: 10.3390/ijerph20064983 (PMC10049230; doi:10.3390/ijerph20064983)
Supplement: Supplementary file 1 [file ijerph-20-04983-s001.zip › Table_S2.pdf]

Table S2. Total length of follow-up in the eventful and non-eventful cases in both groups.

Presence of tendon disease (eventful)	Liver-disease	Non-liver-disease	p-Value
Positive	38.8±28.2	33.1±31.1	0.024
Negative	74.3±25.1	75.6±25.6	<0.001
