# Supplementary material for: Tendon Disorders in Chronic Liver Disease: A Retrospective Cohort Study in Taiwan
Source: Int J Environ Res Public Health. 2023 Mar 12;20(6):4983. doi: 10.3390/ijerph20064983 (PMC10049230; doi:10.3390/ijerph20064983)
Supplement: Supplementary file 1 [file ijerph-20-04983-s001.zip › Table_S3.pdf]

Table S3. The proportion of tendon disorder in liver disease by the codes for liver cirrhosis.

	Patients, n(%)	Eventful, n(%)	p-value
Liver disease	20479(100)	348 (1.7)	<0.001
Non-cirrhosis	11539(56.3)	164 (1.4)	
Cirrhosis	8940(43.7)	184 (2.1)	
