# Supplementary material for: Tendon Disorders in Chronic Liver Disease: A Retrospective Cohort Study in Taiwan
Source: Int J Environ Res Public Health. 2023 Mar 12;20(6):4983. doi: 10.3390/ijerph20064983 (PMC10049230; doi:10.3390/ijerph20064983)
Supplement: Supplementary file 1 [file ijerph-20-04983-s001.zip › Table_S4.pdf]

Table S4. The distribution of viral hepatitis among patients with tendon disorder in the liver-disease group.

Tendon disorder	HBV	HCV	HBV+HCV	None	Total	p-Value
Yes	1684 (8)	838 (4)	2522 (6)	1684 (8)	2522 (6)	
No	2045 (10)	1587 (8)	3632 (9)	2045 (10)	3632 (9)	
